# Assessing Effectiveness of Passive Exoskeletons and Tool Selection on Ergonomic Safety in Manhole Cover Removal

**DOI:** 10.3390/s25072027

**Published:** 2025-03-24

**Authors:** Xun Wang, Ali Golabchi, Maryam Shakourisalim, Karla Beltran Martinez, Zeinab Estaji, Sarah Krell, Mahdi Tavakoli, Hossein Rouhani

**Affiliations:** 1 Department of Mechanical Engineering, University of Alberta, Edmonton, AB T6G 2R3, Canadamshakour@ualberta.ca (M.S.); beltranm@ualberta.ca (K.B.M.); estaji@ualberta.ca (Z.E.); 2 Department of Civil and Environmental Engineering, University of Alberta, Edmonton, AB T6G 2R3, Canada; alireza1@ualberta.ca; 3 EWI Works International Inc., Edmonton, AB T6E 3N8, Canada; 4 EPCOR, Edmonton, AB T5H 0E9, Canada; sarah.krell01@gmail.com; 5 Department of Electrical and Computer Engineering, University of Alberta, Edmonton, AB T6G 2R3, Canada; mtavakol@ualberta.ca; 6 Glenrose Rehabilitation Hospital, Edmonton, AB T5G 0B7, Canada

**Keywords:** ergonomic risk assessment, industrial exoskeleton, low back pain

## Abstract

Manual material handling, a common practice in various industries, often involves moving or lifting heavy objects, placing significant physical strain on workers, especially in the lower back. A prime example is manhole cover removal, which typically requires handling heavy weights, potentially leading to lower back muscle strain. This study investigates the effectiveness of a passive exoskeleton in reducing ergonomic risks during manhole cover removal. Twenty able-bodied workers participated, performing the task with and without extractor tools in the field. Techniques such as surface electromyography and inertial measurement units were employed to measure muscle activity and body posture using the Rapid Entire Body Assessment (REBA). This study compared muscle activities and REBA scores under different conditions: manually lifting covers, using an in-house lever tool, and using a sledgehammer and a pick bar tool named Jake, both with and without an exoskeleton. Results revealed that the in-house Lever tool was the safest and most efficient method, resulting in the lowest muscle activities and REBA scores, regardless of exoskeleton use. Interestingly, the exoskeleton significantly reduced muscle strain when using the Jake tool. These findings indicate that while the Lever tool is optimal for this task, passive exoskeletons can effectively lower ergonomic risks associated with more physically demanding tools.

## 1. Introduction

Low back pain (LBP) is a prevalent work-related musculoskeletal disorder (WMSD), particularly in physically demanding occupations such as construction, utilities, and city maintenance [1,2]. Mild, occupationally induced LBP symptoms can be alleviated by eliminating awkward postures, reducing the workload, and performing athletic stretching on the job site [3]. However, manual material handling remains a leading cause of LBP due to heavy lifting, awkward postures, and overexertion [4,5,6].

**Figure 1:**
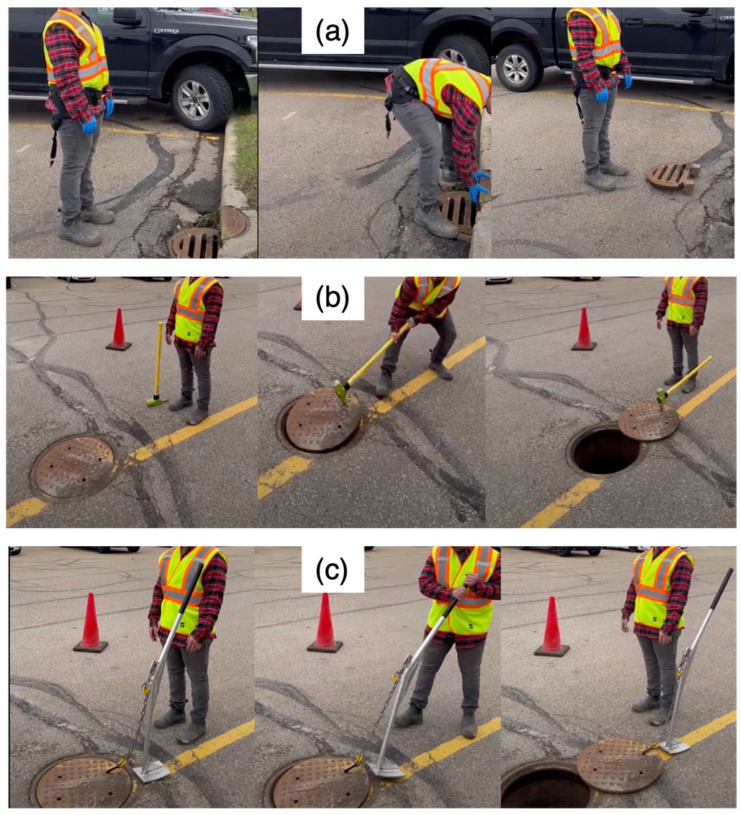
A utility worker removing an 18.1 kg (40 lbs) cover from the manhole manually, without any tools (**a**); the worker removing a 56.7 kg (125 lbs) cover using a Jake tool (**b**) and removing a 56.7 kg cover using a Lever tool (**c**).

Manual material handling is a common task in various industries that significantly strain workers’ lower backs. Surveys show that 25% of workers who perform manual material handling experience back pain lasting more than 7 days annually, with 14% requiring medical attention and 10% needing time away from work. Moreover, back issues account for 38.5% of all work-related musculoskeletal disorders. The Bureau of Labor Statistics notes that more than one million workers suffer back injuries each year, representing one-fifth of all workplace injuries or illnesses in the United States [7,8]. Within this context, manhole cover removal stands out as a particularly demanding task commonly encountered by utility maintenance workers. This activity exemplifies the challenges of manual material handling in the field. Many utility maintenance workers are required to move or lift manhole covers. There are different types of covers, such as catch basin (two-part curb fitting cover) and manhole cover, which can be light or heavy depending on the roadways. These covers are usually made of cast iron and have weights of up to 115 kg [9] to make them durable and resistant to damage and vandalism. These covers are also flush with the ground, resting on top of a frame, which requires the workers to lift them, which can potentially be unsafe if not performed correctly due to their weight and lack of safe latching points for the tools, which can lead to awkward postures and overexertion [10]. Workers typically handle these covers manually (Figure 1a). However, when heavier covers need to be handled, workers often use tools such as a Jake tool, a combination of a sledgehammer and a pick bar (Figure 1b). The covers dragged with a Jake tool weigh over 54.4 kg (120 lbs), which can overexert the lower back’s musculoskeletal system. Moreover, the Jake tool requires repeated hammering and pulling motions, which can strain the lower back and upper limbs. This tool is traditionally used in utility maintenance and is commonly employed for manhole cover removal. However, despite its widespread industrial application, there are no studies assessing its ergonomic impact or biomechanical effectiveness in manual handling tasks. Workers frequently face hazardous conditions due to the excessive weight of the objects they handle and the adoption of unsafe postures. This situation results in disproportionate rates of work-related LBP [11,12].

To reduce the load and change the working posture to an ergonomically favorable posture, a Lever tool with a metal bar, a pedal, and an adjustable chain with a hook at the end can be used (Figure 1c). When a Lever tool is used, the working posture and load on lower back muscles are improved. However, the Lever tool used in this study was an in-house-developed device, designed specifically to mitigate ergonomic risks associated with manhole cover removal. As such, no published academic studies currently exist on its effectiveness, making this study a first step in evaluating its impact on worker biomechanics. Although this tool was designed to improve lifting posture, it requires high exertion at the initial lift and may not be ergonomically optimized for all working conditions. Although a Lever tool is conceptually safer than manual handling or a Jake tool, its efficiency in reducing the ergonomic risk of handling manhole covers has not yet been fully characterized.

In addition to implementing tools, many employers have applied innovative tools and machinery and used personal protection equipment (PPE) to ensure workers’ safety. Recently, passive and powered exoskeletons have been introduced as assistive devices in several industries, including the automotive, construction, oil and gas, and utilities sectors [13]. The prices of industrial active and passive exoskeletons vary depending on their design, functionality, manufacturing process, and market demand [3]. Given their lower cost and convenience of being less heavy and not being battery-dependent, passive exoskeletons have been more widely implemented in industries than powered ones [3]. Exoskeletons are designed to support various body parts, including the shoulder, trunk, leg, and full body, depending on the working posture and load [14,15]. However, their performance depends on the differences in structure and working mechanism, and there is no guarantee that they will be efficient for a particular activity [14]. Several types of passive exoskeletons have been developed for different physical tasks. Their effectiveness has been evaluated in reducing muscle activity by 35–38% in the upper limb [16], by 25% in the leg [17], and up to 80% in the lower back [14], as well as in various industrial scenarios. However, exoskeletons can also induce compensation by the antagonist muscle or spinal imbalance due to posture changes [18]. Given their recent introduction to industrial applications, the efficacy of passive exoskeletons for most real-world jobs involving various static and dynamic postures, such as manhole cover removals in utility maintenance, has not been thoroughly investigated.

The purpose of this study was to assess the effectiveness of passive exoskeletons and various tools on ergonomic risk when performing the manhole cover removal task and suggest safe, cost-effective, and practical methods for this task. In a previous study, we showed that ergonomic risk assessment for manhole cover removal can lead to significantly different results when non-workers simulate the task in a research laboratory with an object similar to a manhole cover compared to real-world scenarios [19]. Therefore, in the present study, we investigated the impact of passive exoskeletons on reducing ergonomic risks. At the same time, utility workers removed manhole covers in the field, manually or with the aid of a Jake or a Lever tool while wearing or not wearing the exoskeleton. We hypothesized that using the tools and the exoskeleton would reduce the ergonomic risk and overexertion.

## 2. Materials and Methods

### 2.1. Study Design and Participants

Twenty able-bodied utility workers (19 males, 1 female, body mass: 78 ± 15 kg, body height: 172 ± 9 cm, age: 37 ± 10 years) with no clinical history of lower back pain up to six months prior to the study, volunteered to participate in the experiments. The number of participants depended on the workers’ availability. The participant group consisted primarily of male workers (19 out of 20), which may influence the generalizability of the findings. The trials were conducted at EPCOR Utilities (Edmonton, Alberta, Canada), and the setup was designed to replicate the tasks performed by utility workers on the job site. None of the participants had prior experience with the exoskeleton; however, they were familiar with the Jake and Lever tools through their regular job duties. This study was approved by the research ethics board of the University of Alberta ID: Pro00109264. Written informed consent for participation and publication of any photos or videos captured during the experiment was obtained from all the participants. A portion of the data collected and used in the present study was previously used to investigate differences between in-lab and in-field ergonomic risk assessment for manhole cover removal using an exoskeleton [19] rather than assessing the impact of the exoskeleton itself on this task.

### 2.2. Passive Back-Support Exoskeleton

**Figure 2:**
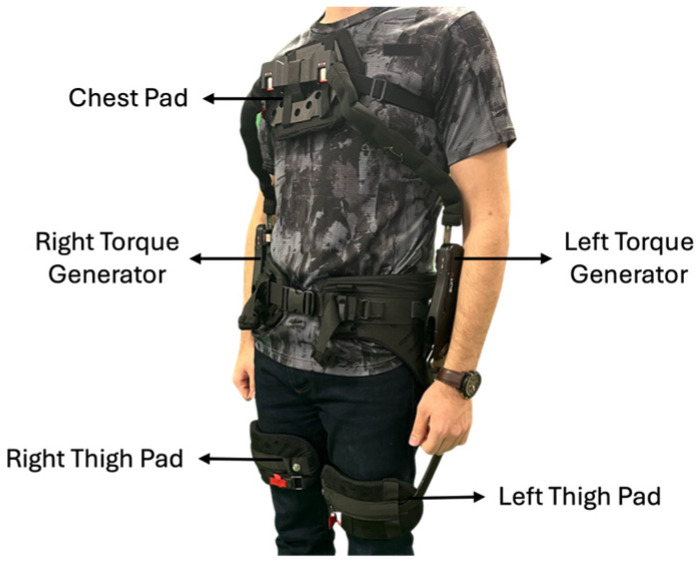
A user wearing the BackX exoskeleton. The exoskeleton supports the lower back by redistributing forces through its rigid frame and contact points at the chest and thighs.

The exoskeleton used in this study was a back-support exoskeleton BackX from SuitX (Emeryville, CA, USA). The BackX has gas springs on the sides to provide trunk extension torque and has support points on the thighs and chest with a joint on the hip (Figure 2). The exoskeleton reduces gravity-induced forces and torques on the lower back by applying a supporting force to the wearer’s chest and thighs, offloading the lower back during stooping, lifting, and reaching tasks. The system activates when the user bends forward, using a passive assistive mechanism to aid movement. The exoskeleton provides bilateral support, distributing forces symmetrically across both sides of the body. This ensures balanced assistance during bending and lifting movements. The exoskeleton has two designed modes: (i) instant mode, in which the support of the exoskeleton is always activated, and (ii) standard mode, in which the exoskeleton gets activated once the user’s trunk bending angle reaches 30 degrees. The user can walk and move freely when the exoskeleton is not engaged. This exoskeleton is designed to reduce the load on the lower back musculoskeletal system while bending forward by reducing forces and torques applied to the lower back. Consequently, the activation of lower back muscles was expected to reduce with the application of the exoskeleton.

### 2.3. Experimental Procedure

#### 2.3.1. Muscle Activity Data Collection

The participants were first familiarized with the experiment and equipped with surface electromyography (sEMG) and inertial measurement unit (IMU) sensors. After sensor placement, maximum voluntary contraction (MVC) tests were conducted to normalize the measured muscle activity following Konrad’s recommendations [20]. For this purpose, participants executed isometric contractions against resistance for each muscle group with verbal encouragement to ensure maximal effort, and the highest EMG amplitude over a 5 s window was identified as the MVC reference value. For back muscles (Latissimus Dorsi and Erector Spinae), normalization followed the protocol established in our previous study, where MVC was determined from a standardized trunk extension task [21]. This approach has been validated for assessing trunk muscle activation during lifting tasks. The highest recorded EMG value from the trials was used as the MVC reference for normalization. Since each participant’s EMG data were normalized to their own MVC values, the use of different MVC determination methods across muscles did not affect within-participant comparisons, ensuring consistency in statistical analysis.

#### 2.3.2. Task Description

Participants were allowed to wear the exoskeleton and perform simple tasks, such as walking and squatting, to get accustomed to the device before the test.

To simulate actual working conditions, the trials were designed based on the suggestions from EPCOR Utilities. To investigate the impact of each tool on reducing ergonomic risks, the trials involved the removal of manhole covers in three ways: for the light cover (18.1 kg), the participants moved the cover manually without using any tools. For the heavier cover (56.7 kg), the participants moved the cover once using the Jake tool and once using the Lever tool (Figure 1). Each trial began and ended with five seconds of standing still to remove the IMUs’ measurement offset and was repeated twice for validity. The loads varied between manual handling and tool-assisted conditions. Thus, the loads were not treated as an independent variable. Instead, comparisons were made within each condition separately to account for the load differences.

**Figure 3:**
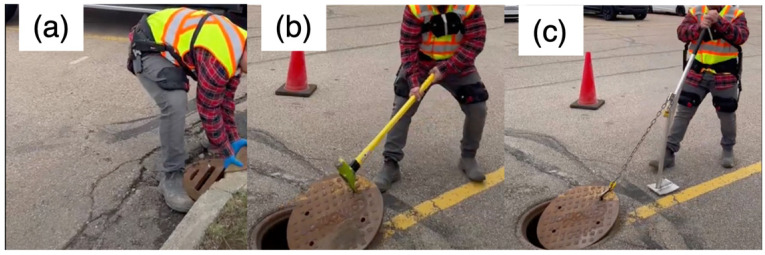
Lifting a manhole cover manually (**a**), with a Jake tool (**b**), and with a Lever tool (**c**) while wearing the BackX exoskeleton.

In order to investigate the impact of wearing an exoskeleton on reducing ergonomic risks, the trials were repeated while wearing an exoskeleton in both standard and instant modes (Figure 3). Table 1 summarizes all the trial conditions. Workers performed tasks using their natural movement strategies, as this study aimed to assess real-world performance rather than impose artificial constraints. While posture was not strictly controlled, all workers had prior job site training on optimized task postures to reduce injury risk. This approach ensured that results reflected typical work conditions and the practical impact of the exoskeleton.

Finally, during and after all trials, the participants’ feedback on the efficiency and comfort of the exoskeleton was recorded. Each participant provided feedback separately to reduce bias in the qualitative data. Also, by applying a concurrent Likert scale alongside simple and open-ended questions, participants were guided to provide a more structured answer.

### 2.4. Measurement and Data Analysis

#### 2.4.1. Muscle Activation Assessment

**Figure 4:**
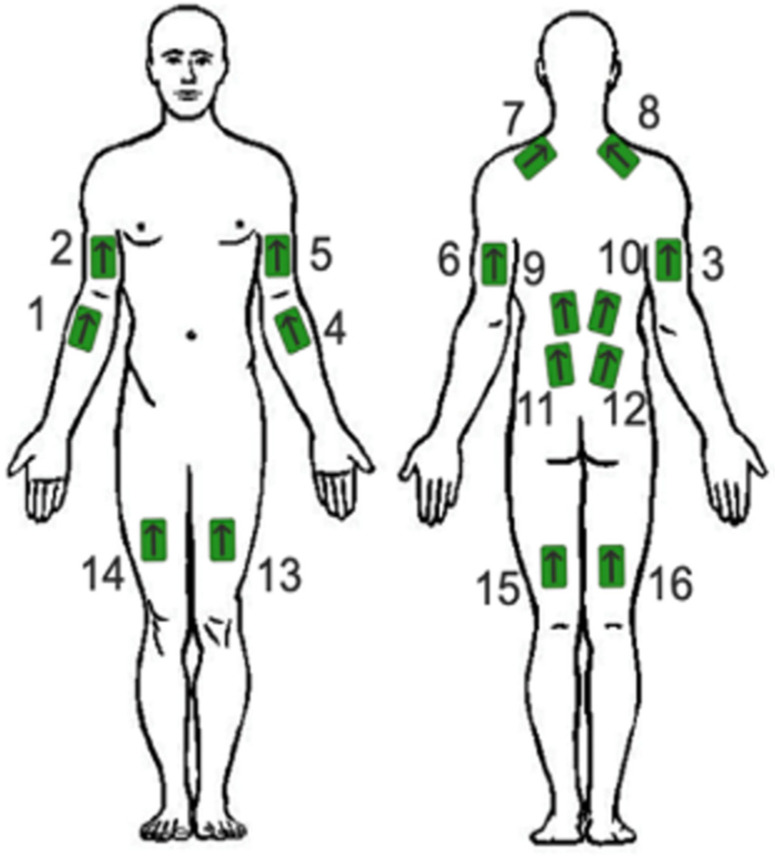
Surface electromyography (sEMG) sensor placement on body muscles: (1) Right Brachioradialis, (2) Right Biceps Brachii, (3) Right Triceps Brachii, (4) Left Brachioradialis, (5) Left Biceps Brachii, (6) Left Triceps Brachii, (7) Left Trapezius middle branch, (8) Right Trapezius middle branch, (9) Left Latissimus dorsi, (10) Right Latissimus dorsi, (11) Left Erector Spinae, (12) Right Erector Spinae, (13) Left Rectus femoris, (14) Right Rectus femoris, (15) Left Biceps femoris, (16) Right Biceps femoris. The arrow on the EMG sensors indicates the axis of the differential electrode pair and should be aligned parallel to muscle fibers to maximize signal quality.

SEMG sensors (Trigno, Delsys Inc., Middlesex County, MA, USA) were used to measure muscle activity during manhole cover removal. The sensors were placed on the participant’s body to record the activity of 16 muscles, as shown in Figure 4, following the SENIAM’s guidelines [22]. These muscles were selected based on their functional involvement in postural control and task execution. The back muscles were included to assess spinal stabilization under exoskeletal support, while lower limb muscles were chosen to evaluate compensatory strategies during movement. These muscles comprised both the lower body, supported by the exoskeleton, and the upper body, which did not interact with the device (Figure 4). The data were recorded at a sampling frequency of 2148.15 Hz using EMGworks acquisition software (version 4.8.0; Trigno, Delsys Inc., Middlesex County, MA, USA). The raw EMG signals of a few participants are presented in the Supplementary Materials. The baseline EMG activity was recorded while participants were standing rather than lying down. This choice was made to establish a functional baseline that accounts for natural postural muscle activity, allowing us to evaluate the relative effect of the exoskeleton when transitioning from a non-task standing condition to task execution. While this approach did not eliminate postural muscle activation, it provided a valid reference point for assessing the exoskeleton’s influence during real-world use. The following steps were taken to process the sEMG recordings:

1. The recorded sEMG signal was filtered using a 4th-order band-pass Butterworth filter with cut-off frequencies of 10 Hz and 500 Hz, as recommended by Konrad [20]. To ensure a stable signal baseline, the mean value of the signal was subtracted prior to further analysis.
2. Full wave rectification was performed.
3. To smooth the rectified signal, a moving average filter with a window length of 500 was applied to the data.
4. The baseline error was rechecked and, if needed, eliminated.
5. The EMG signal amplitude was normalized by an MVC process for each muscle according to Konrad’s recommendation [20], except for the back muscles (Latissimus Dorsi and Erector Spinae), which followed the procedure introduced previously by our group [21].
6. The RMS of the normalized sEMG amplitude was calculated during the working period.

#### 2.4.2. Posture-Based Risk Assessment

**Figure 5:**
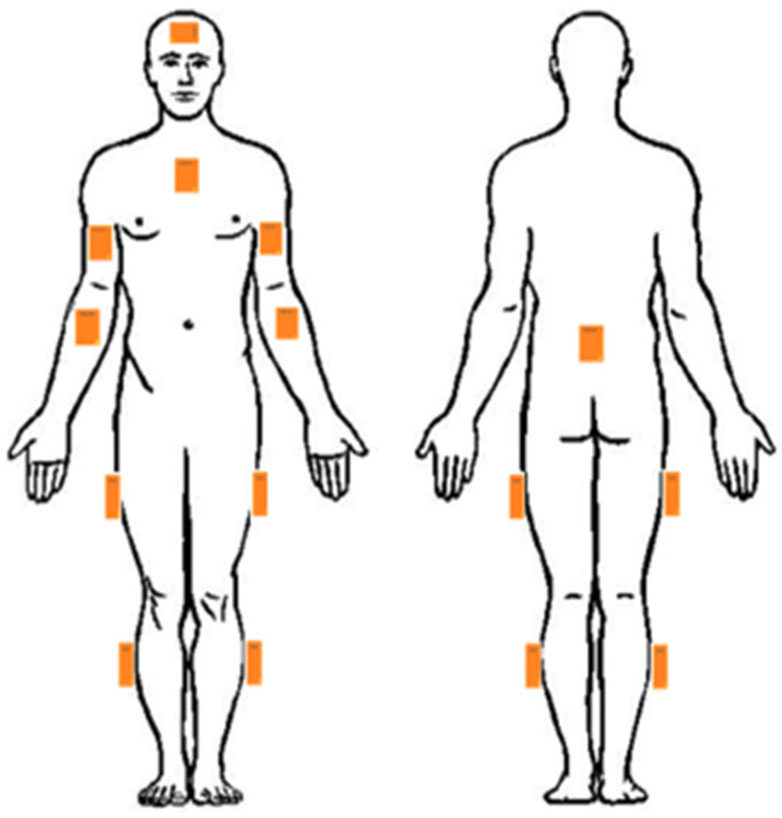
IMU sensor placement on the body segment. The orange boxes represent IMUs placed on the forehead, chest (over sternum), pelvis (over sacrum), upper arms, forearms, thighs, and shanks.

In the literature, posture-based risk assessment tools such as Rapid Entire Body Assessment (REBA) or Rapid Upper Limb Assessment (RULA) [23] are used to analyze the WMSD risk in general tasks based on body posture and joint angles. The authors previously validated the accuracy of REBA scores for a manual handling task obtained by wearable IMUs [24,25]. The present study used wearable IMUs (MTws, Xsens Technologies, The Netherlands) to obtain the REBA score during the in-field manhole cover removal task. Figure 5 shows the placement of IMUs on the participant’s body. A sensor-to-body calibration procedure suggested by Nazarahari et al. [26,27] was performed to obtain physiologically meaningful joint angles. The 3D orientation of body segments and 3D joint angles were obtained using IMUs and previously validated algorithms [28]. Finally, the REBA scores, as described in [23], were calculated using the joint angles obtained by the IMUs.

#### 2.4.3. Usability and Wearer Comfort

**Figure 6:**
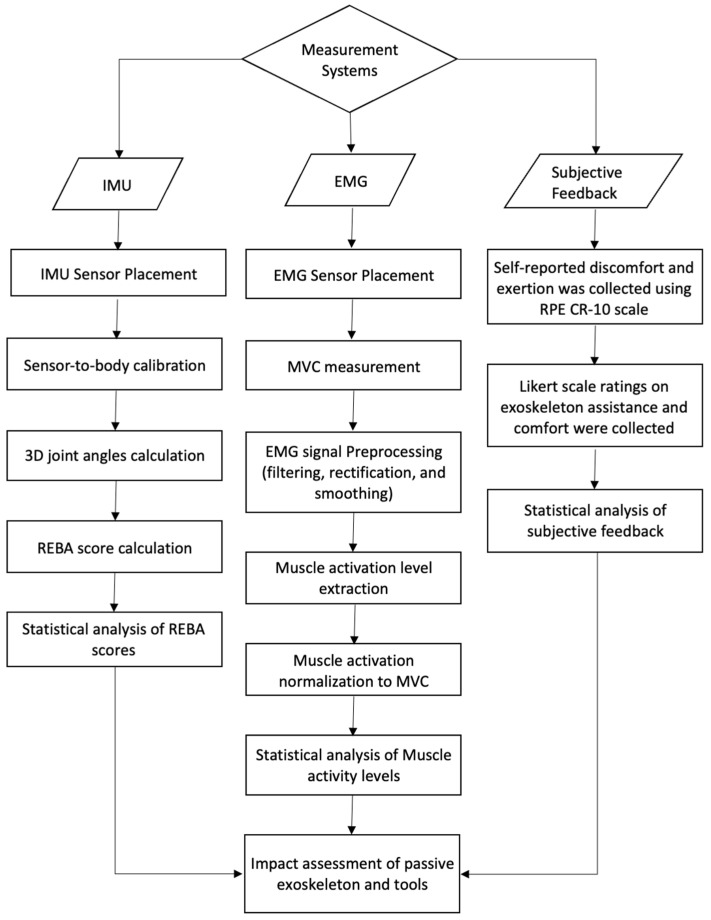
Flowchart outlining the measurement and data analysis steps. The process includes three sets of collected data: IMU-based posture assessment, EMG-based muscle activation analysis, and subjective feedback collection. Data from these sources are processed to estimate ergonomic risk using REBA scores, muscle activation levels, and self-reported exertion ratings.

To assess the effectiveness and comfort of the exoskeleton, a rated perceived exertion (RPE CR-10) scale was employed, which allowed the participants to evaluate the level of work intensity required for each task [29]. The RPE scale was shown to participants as presented in [30], where 1 is a very light activity: hardly any exertion and 10 is a maximum effort activity: it feels almost impossible to keep going. Additionally, a questionnaire was collected at the end of the test to gather feedback from participants on using the exoskeleton and tools and their perception of the level of assistance provided in various body parts. Figure 6 shows a diagram of the measurement and data analysis steps.

### 2.5. Statistical Analysis

Statistical data analysis was performed to evaluate the effectiveness of the tools and the exoskeleton in the manhole cover removal task. The statistical analysis was conducted separately for each condition with and without the exoskeleton to clearly assess the exoskeleton’s impact when each tool was used. As the job site regulations mandated the use of tools for heavier covers, direct comparisons between manual and tool-assisted conditions were not appropriate.

The independent variables were the use of the exoskeleton and tools, while the dependent variables included (i) the muscles’ activity (characterized by the normalized sEMG amplitude), (ii) body posture (characterized by the REBA score), and (iii) feedback from participants. Each dependent variable was averaged over both trials of each participant in each tool or exoskeleton usage condition. To evaluate the tools’ effectiveness without wearing the exoskeleton, the manhole cover removal task was performed with and without using tools (Jake and Lever), and the dependent variables for all participants in each condition were compared. Moreover, to evaluate the effectiveness of the exoskeleton, the manhole removal task was performed (manually and tool-assisted) with and without wearing the exoskeleton, and the dependent variables for all participants in each condition were compared. Additionally, both exoskeleton modes (standard and instant) were evaluated during manhole cover removal while wearing the exoskeleton.

The distribution of the collected data was not normal based on the Shapiro–Wilk (S-W) test. Therefore, the Wilcoxon signed-rank test (significance level: 5%) was used to assess if there were any significant differences within the dependent variables for all paired comparisons [31]: (i) comparing dependent variables when the tasks were performed manually and with each tool and (ii) comparing dependent variables when the tasks were performed without an exoskeleton and with an exoskeleton.

## 3. Results

### 3.1. Impact of Using the Jake and Lever Tool Without an Exoskeleton on Muscle Activation During Manhole Cover Removal

**Figure 7:**
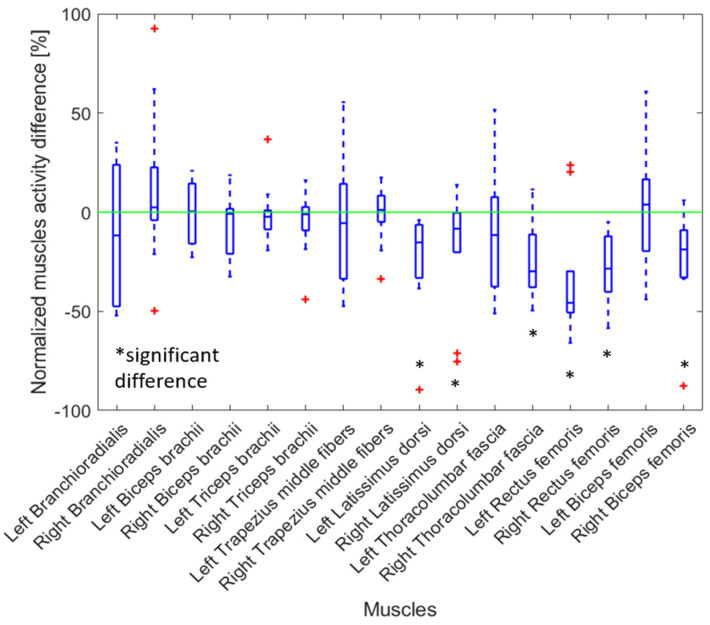
The relative change in muscle activity when a Jake tool was used compared to when a Lever tool was used to remove a manhole cover. The results for all participants are presented as boxplots. A positive percentage shows increased muscle activity when a Lever was used. Red crosses indicate an outlier. Black asterisks indicate a significant difference with zero with *p*-values < 0.05.

The muscle activity when using the Jake tool was greater for most muscles compared to when a Lever tool was used (Figure 7). There was a significant increase in muscle activation in the left and right latissimus dorsi (24.1% and 19.4%, respectively), right thoracolumbar fascia (25.5%), left and right rectus femoris (32.8% and 28.7%, respectively), and right biceps femoris (22.4%).

### 3.2. Impact of Using the Exoskeleton on Muscle Activity During Manhole Cover Removal Manually

**Figure 8:**
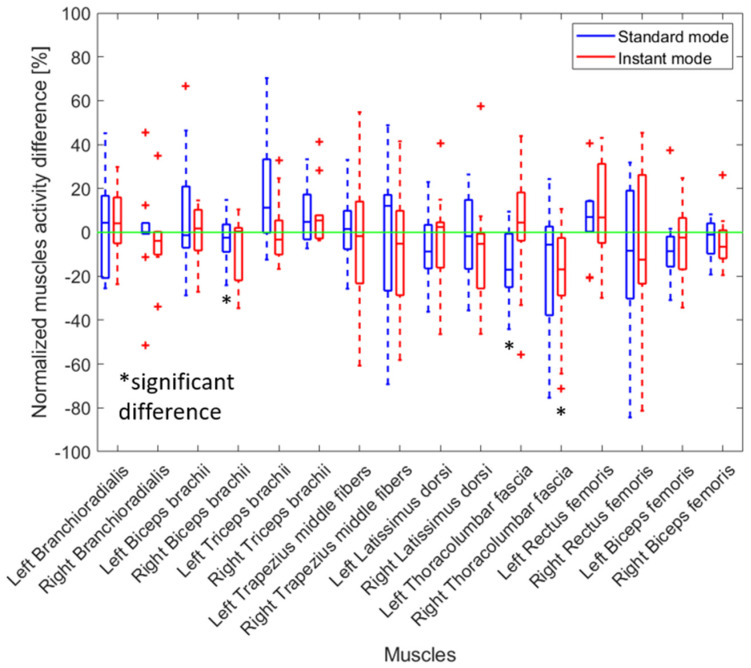
The relative change in muscle activity when a manhole cover was removed manually (no tools) with an exoskeleton (instant and standard mode) compared to not wearing an exoskeleton. The results for all participants are presented as boxplots. A positive percentage shows an increase in muscle activity when the exoskeleton was worn. Red crosses indicate an outlier. Black asterisks indicate a significant difference with zero with *p*-values < 0.05.

The standard mode of the back-support exoskeleton significantly increased the muscle activity of the left triceps brachii by 18.7% among participants and significantly decreased the activity of the left thoracolumbar fascia by 15.0% (Figure 8). When the exoskeleton was in instant mode, it significantly decreased the activity of the right thoracolumbar fascia by 21.5%.

### 3.3. Impact of Using the Exoskeleton on Muscle Activity When a Jake or Lever Tool Was Used

**Figure 9:**
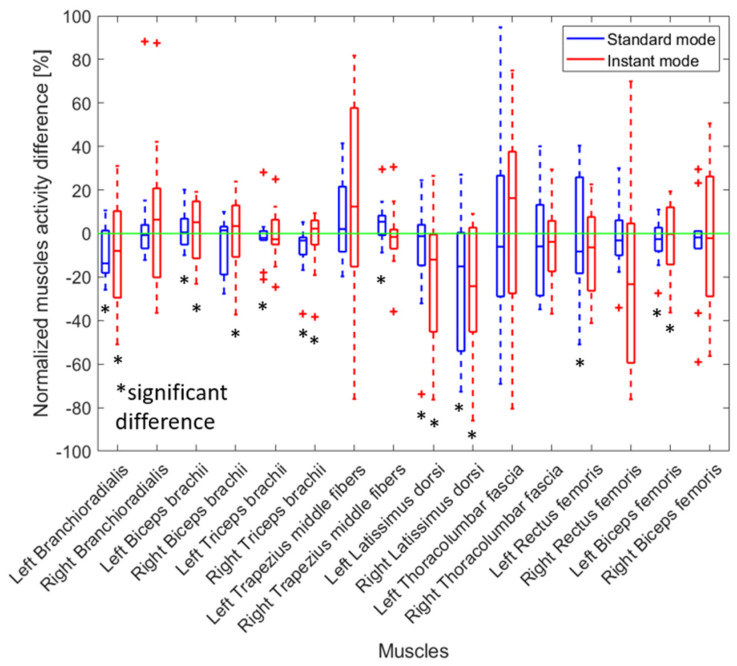
The relative change in muscle activity when a manhole cover was removed using a Jake tool with an exoskeleton (instant and standard mode) compared to not wearing an exoskeleton. The results for all participants are presented as boxplots. A positive percentage shows an increase in muscle activity when the exoskeleton was worn. Red crosses indicate an outlier. Black asterisks indicate a significant difference with zero with *p*-values < 0.05.

Wearing the back-support exoskeleton while removing manhole covers using a Jake tool significantly reduced the activity of many muscles in both instant and standard modes (Figure 9). The largest change was observed for the left and right latissimus, with reductions of 10.2% and 17.6% (left) and 21.7% and 24.0% (right) in standard and instant modes, respectively. Although the activity of lower limb muscles, such as the left biceps femoris, was reduced, the activity of other muscles, such as the biceps brachii, increased with the use of the back-support exoskeleton.

**Figure 10:**
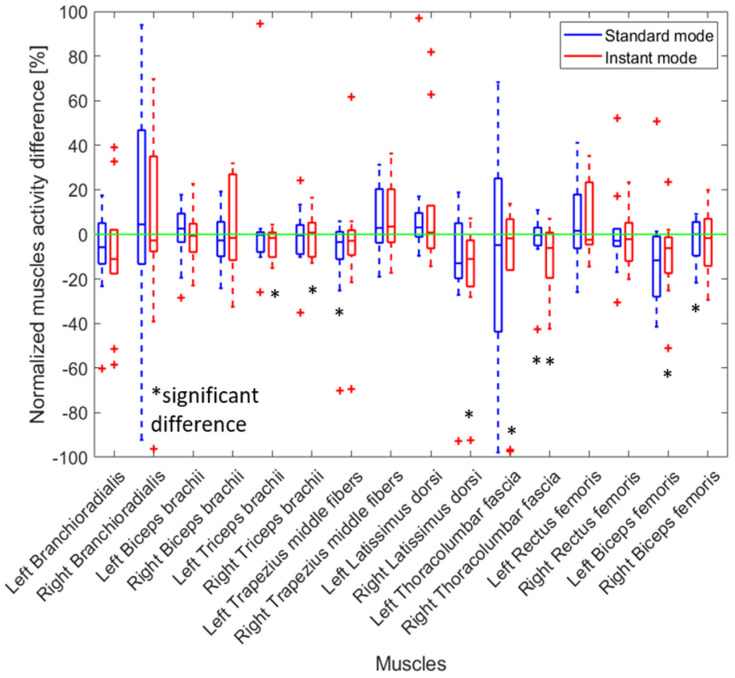
Distribution among participants showing the relative change in muscle activity when a manhole cover was removed using a Lever tool with an exoskeleton (instant and standard mode) compared to not wearing an exoskeleton. A positive percentage shows an increase in muscle activity when the exoskeleton was worn. Red crosses indicate an outlier. Black asterisks indicate a significant difference with zero with *p*-values < 0.05.

When employing the Lever tool to remove the manhole covers, the use of the back-support exoskeleton led to a significant decrease in muscle activity, especially in the instant mode (Figure 10). Particularly, there was a reduction of 19.2% and 9.7% of muscle activities in left and right thoracolumbar fascia and a reduction of 9.6% and 3.2% of muscle activities in left and right bicep femoris, respectively, when the exoskeleton was used in the instant mode.

### 3.4. Impact of Exoskeleton on the Body Posture During Manhole Cover Removal

**Figure 11:**
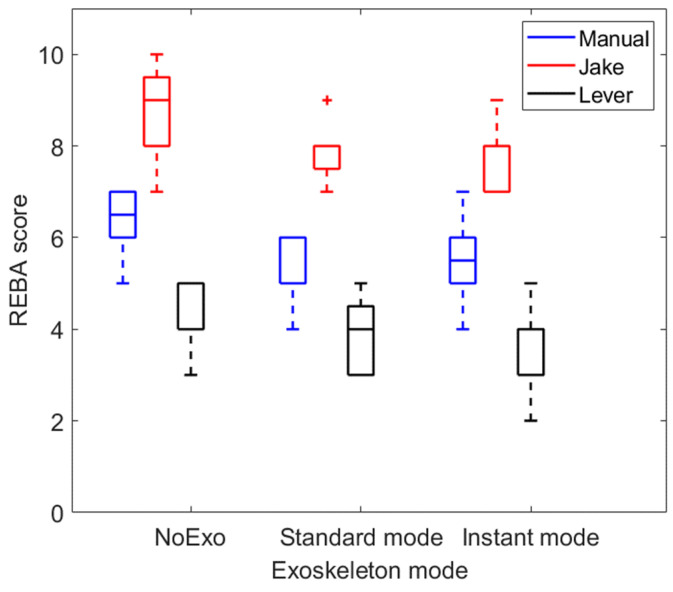
The REBA scores while removing a manhole cover using different tools and with and without an exoskeleton (in both standard and instant modes). The results for all participants are presented as boxplots. Red crosses indicate an outlier.

The use of exoskeleton in both instant and standard modes resulted in a slight improvement in the body posture (characterized by REBA score) during manhole cover removal manually, with the Jake tool, and with the Lever tool. To study the impact of the exoskeleton on the body posture, the differences in REBA scores were also tested by the Friedman test. The mean REBA score decreased from 6.4 to 5.3/5.4 (standard/instant) for manual handling, from 8.9 to 7.9/7.7 (standard/instant) for the cover removal with the Jake tool, and from 4.5 to 3.9/3.8 (standard/instant) with the Lever tool, as shown in Figure 11. In the Friedman tests for the three conditions, we obtained *p*-values of less than 0.05 when using the exoskeleton. However, no significant difference was observed between the instant and standard modes of the exoskeleton, with a minimum *p*-value equal to 0.21. Additionally, the use of a Lever tool resulted in a significantly smaller REBA score compared to the use of a Jake tool or removing the manhole manually (*p*-value < 0.05), indicating the lowest ergonomic risk.

### 3.5. Questionnaire

**Figure 12:**
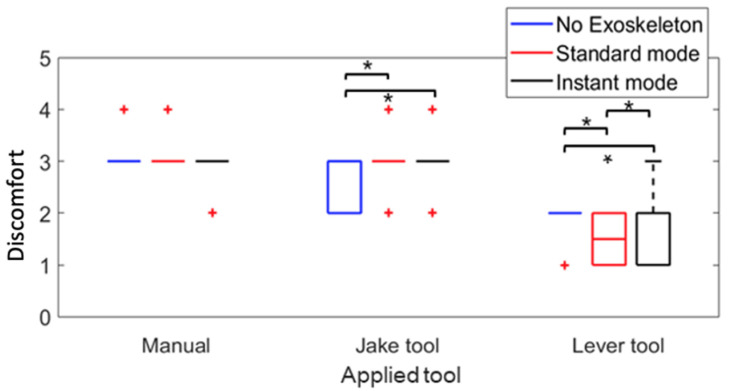
Self-reported user feedback scores on discomfort using RPE scores on tasks using different methods with and without the exoskeleton. The results for all participants are presented as boxplots. Black asterisks indicate a significant difference with *p*-values < 0.05 for the Wilcoxon signed-rank test. Red crosses indicate an outlier.

The outcomes presented in Figure 12 illustrate the participants’ self-reported feedback on the task discomfort levels using a scale of 1 to 10. The feedback was gathered both with and without the exoskeleton and for each tool (Manual, Jake, and Lever). The statistical significance of the results was evaluated using the Friedman test. The impact of wearing the exoskeleton on task discomfort depended on the tools used. When using the Lever tool, the exoskeleton significantly reduced the task discomfort compared to when an exoskeleton was not used (*p* < 0.05). When using the Jake tool, the exoskeleton significantly increased the task discomfort compared to when an exoskeleton was not used (*p* < 0.05). The use of an exoskeleton did not affect the discomfort level when the task was performed manually. The differences in reported discomfort across conditions may be due to variations in movement constraints between tools. For example, when using the Jake tool, participants performed hammering and pulling motions, which required greater trunk and hip mobility. The exoskeleton, designed to provide postural support, may have restricted these natural movements, leading to increased discomfort. In contrast, using the Lever tool involved a more upright posture with reduced forward bending, aligning more with the exoskeleton’s support mechanism. This likely contributed to the perceived reduction in discomfort in the Lever condition.

**Figure 13:**
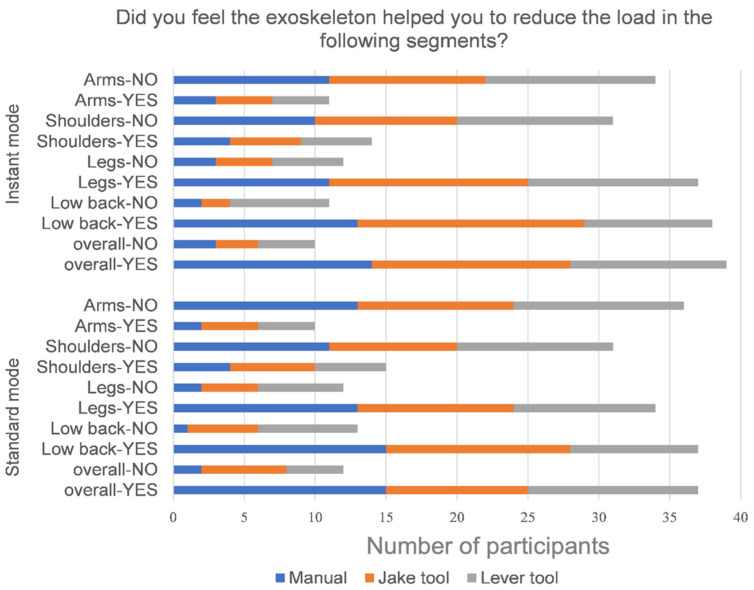
Self-reported user feedback on reduction in load when using the exoskeleton.

The participants were also asked about their perception of the exoskeleton assistance while using each tool during the tasks. The results were then subjected to a statistical analysis using the Friedman test, revealing that all *p*-values were less than 0.05. This indicates that the differences in the feedback regarding the exoskeleton’s assistance with each tool were all statistically significant. Around 80% of participants were confident that the exoskeleton could help reduce the working load on the leg and lower back, but not on the shoulder and arm (Figure 13). Also, they felt that both exoskeleton modes reduced the overall workload on the body.

## 4. Discussion

This study investigated the effectiveness of a passive back-support exoskeleton in reducing ergonomic risks during the manhole cover removal procedure. The task was performed manually using a Jake tool and a Lever tool. To this end, we compared the muscle activity characterized by sEMG sensors and the REBA score characterized by IMUs in different scenarios. It was the first time that such physiological measurements were conducted in a real-world environment and for workers to assess the effectiveness of a passive exoskeleton for manhole cover removals.

### 4.1. Comparison with Previous Research and Real-World Applications

Our findings align with previous research on passive exoskeletons. Schwartz et al. [32] found that passive lumbar exoskeletons effectively reduced lower back muscle activity during static lifting tasks. Govaerts et al. [33] showed that passive exoskeletons lowered physical strain in manual handling tasks. Similarly, Ahn et al. [34] observed muscle activation reductions when using a passive exoskeleton in laboratory-controlled manual lifting tasks. These studies highlight the potential of passive exoskeletons to mitigate musculoskeletal strain in demanding jobs.

Our study uniquely focused on real-world applications, specifically manhole cover removal, a demanding and previously underexplored task in exoskeleton research. We provided practical insights into how these devices function in the field by evaluating the interaction between passive exoskeletons and existing industrial tools (Jake and Lever tools). Unlike laboratory studies, our research captured the natural variability of worker movement strategies and tool use, making the findings more applicable to real-world industry job sites.

### 4.2. Exoskeleton’s Effect on Removing the Manhole Cover Manually

Applying a back-support exoskeleton was expected to lower the ergonomic risks and muscle load, especially on the lower back muscles [12,35]. However, when this device was used to perform the manual lifting task, we did not observe a significant difference in the muscle activity for most body parts. This lack of effectiveness of the exoskeleton could be due to (1) the high inter-participant variability of body motion and maneuver for manhole cover removal or (2) a lack of familiarity of the workers with the use of the exoskeleton and restriction on their preferred body movement by the device.

The observed high inter-individual variability in Figure 7 was likely due to differences in movement strategies and muscle engagement. The inter-participant differences contribute considerably, as muscle activation patterns vary based on individual biomechanics, movement strategies, and adaptation to the exoskeleton. Also, manually removing the manhole cover requires complex, multi-joint coordination, resulting in inconsistent muscle recruitment across trials.

### 4.3. Exoskeleton’s Effect on Removing the Manhole Cover Using the Lever Tool

When the passive back-support exoskeleton was used to support the manhole cover removal task with a Lever tool, there was a reduction in muscle activity in the lower back and lower limbs. The activity of the right latissimus dorsi (in the lower back region) reduced by 14.8% when the exoskeleton was in standard mode and by up to 19.2% in instant mode. Additionally, the addition of the exoskeleton did not change the REBA scores significantly. The difference in the effectiveness of the exoskeleton, when combined with the Jake and Lever tools, could be due to the body posture required for using these tools. Trunk-bending tasks involve a high load on the lower back muscles and thus benefit most from using a back-support exoskeleton [36,37]. In summary, the effectiveness of a passive back-support exoskeleton depends highly on the body posture required for the task [38,39].

### 4.4. Exoskeleton’s Effect on Removing the Manhole Cover Using the Jake Tool

When a Jake or Lever tool was used, workers’ movement patterns were more consistent (based on the researchers’ observations), leading to a more uniform influence of the exoskeleton across participants. Notably, the exoskeleton significantly reduced muscle activity when combined with the Jake tool, with the right latissimus dorsi showing a 21.7% reduction in standard mode and 24.0% in instant mode.

The greater reduction in muscle activation when using the Jake tool, compared to the Lever tool, is due to differences in posture and force application. The Jake tool requires more forward bending, increasing the moment arm of the upper body and placing higher mechanical loads on the lower back. In contrast, the Lever tool allows for a more upright posture, reducing strain on the lumbar spine.

Since the Jake tool places greater demands on the lower back, the exoskeleton provides more noticeable support by redistributing forces away from the spine. This explains why the exoskeleton was more effective in reducing lower back muscle activation when used with the Jake tool. This effect was less pronounced when using the Lever tool, likely because its mechanism already improves posture and reduces forward bending, thereby lowering baseline muscle activation levels even before adding the exoskeleton. Furthermore, adding an exoskeleton improved the body posture of the workers based on the REBA score (Figure 11).

Muscle activation asymmetries observed in this study can be attributed to natural biomechanical differences between dominant and non-dominant limbs, as well as individual variations in movement strategy and task execution. During manual handling tasks, workers may favor one side of the body, leading to uneven muscle recruitment. Additionally, the nature of the Jake and Lever tools requires asymmetric force application, further contributing to differences in activation patterns between the left and right sides. While these asymmetries are expected in real-world manual handling tasks, our statistical analysis ensures that significant trends in muscle activation are still identifiable.

### 4.5. Exoskeleton’s Effect on the User Feedback

The participants’ feedback also demonstrated that wearing the exoskeleton significantly reduced their perception of discomfort associated with the task, except for lifting the cover manually. Furthermore, over 75% of the participants felt confident in the effectiveness of the exoskeleton in reducing the workload of lower back and leg segments during manhole cover removal in all situations.

The findings of this study highlighted the complex relationship between objective biomechanical improvements and subjective perceptions of discomfort and exoskeleton assistance. While both EMG data and REBA scores indicated that the exoskeleton effectively reduced lower back muscle activity and improved posture, participant feedback did not always align with these ergonomic benefits, suggesting that individual perception, personal biases, and task-specific experiences influenced their responses.

For example, muscle activity data showed that the exoskeleton significantly reduced latissimus dorsi activation, particularly when used with the Jake tool. However, participants did not report a significant reduction in discomfort when using the Jake tool with the exoskeleton. This discrepancy may be due to the intense force application and repetitive upper body engagement required by the Jake tool, which could be influenced by the exoskeleton’s lumbar support.

In contrast, when using the Lever tool, both subjective feedback and objective measures aligned more closely. The Lever tool inherently improved posture and reduced lower back strain, so participants may have felt the exoskeleton’s effects more clearly.

Additionally, survey results indicated that 80% of participants believed the exoskeleton reduced workload on the lower back and legs but not on the arms and shoulders. This aligned with EMG results, which showed increased upper limb activation in some conditions, likely due to compensatory or restricted motion strategies.

### 4.6. Jake Tool Versus Lever Tool Effect on Removing the Manhole Cover

An additional comparison was performed to assess which tool (Jake or Lever) was more suitable for removing manhole covers. We observed that the muscle activities of the lower limb (i.e., rectus femoris and biceps femoris) and lower back (i.e., latissimus dorsi and thoracolumbar fascia) were significantly smaller (between 20% and 32%) when a Lever tool was used compared to when a Jake tool was used (Figure 6). Also, the workers had a standing posture with a lower level of risk (characterized by the REBA score) when the Lever tool was used, with a score of 4.5 compared to the Jake tool, which had a score of 8.9 without the exoskeleton. Additionally, participants felt less difficulty (RPE scores reduced by 1 or more) lifting the manhole cover with a Lever tool, as reflected by their self-reported feedback. Thus, if the worker is not wearing a back-support exoskeleton, it is safer to use the Lever tool compared to the Jake tool.

### 4.7. Limitations

Some of the limitations of this study include the following: (1) While muscle activity (recorded by sEMG), overall body posture (expressed via REBA score), and users’ feedback were integrated into the experiments, other factors such as energy expenditure may impact the exoskeleton’s efficiency and must be studied in the future. In addition, future studies should investigate the impact of passive exoskeletons and tools on body joints’ kinematics and kinetics and their association with the risk of WMSDs. (2) This study allowed participants to perform manhole cover removal naturally, reflecting real-world conditions. However, each participant has unique movement strategies that may have influenced the results. Future studies could use motion capture or wearable sensors to quantify these variations and assess their interaction with exoskeleton assistance more precisely. (3) The findings are limited to a specific type of exoskeleton and in the future, the efficiency of other exoskeletons should be investigated. (4) Larger numbers of participants and trials per participant are needed for a more robust result. (5) Factors such as layers of work clothing, weather conditions (especially in the winter), and site challenges (i.e., traffic) can add layers of complexity, but were not included in this study. (6) For safety considerations, there existed a variance in the weight of the manhole cover between manual removal and removal with tools. This variance could result in inconsistencies when comparing the conditions. (7) Finally, there was only 1 female participant compared to 19 males. The sex of the participants in this study depended on the workers’ availability. However, given the musculoskeletal differences between sexes, it is possible that this led to inconsistencies in the results.

This study used REBA as a standardized and widely accepted method for ergonomic risk assessment, as it provides a practical and industry-relevant evaluation of posture-based risk. The analysis focused on ergonomic risk scores, muscle activity, and subjective feedback rather than detailed joint-level movement data. While this approach aligns with workplace evaluation practices, previous research has explored ergonomic risk quantification using joint angles, especially for fatigue-related ergonomic risks [25,40]. Future studies could investigate how joint movement patterns change with tool use and exoskeletons to enhance posture-based risk assessments further.

In this study, we did not compare conditions with different weights against each other; rather, we analyzed conditions where the same weight was used to maintain consistency in our ergonomic assessment. Our focus was on understanding how passive exoskeletons and tools influenced posture-based ergonomic risk (REBA), muscle activation, and subjective perception. However, future research should explore the direct impact of load variations on ergonomic risk and physiological responses to provide a more detailed evaluation of task demands.

Additionally, given the high inter-participant variability in movement strategies, especially during manual manhole cover removal, future studies should use data-driven methods, such as clustering techniques, to classify different movement patterns. This approach can provide a better understanding of how variability affects ergonomic risk and the effectiveness of passive exoskeletons. Identifying these differences may help improve ergonomic interventions and optimize the support provided by assistive devices.

## 5. Conclusions

Our study evaluated the effectiveness of a passive back-support exoskeleton during manhole cover removal, comparing its impact across manual methods, the Jake tool, and the Lever tool. The results demonstrated that the exoskeleton significantly reduced lower back muscle activity, mainly when used with the Jake tool, where we observed reduced activation of the latissimus dorsi and improved posture scores.

This study’s findings emphasized that the exoskeleton’s effectiveness highly depends on the body posture of the worker while removing the manhole cover. The results showed that the Lever tool exhibits significantly less muscle activity on the lower back (with or without exoskeleton) and a lower REBA score compared to the results revealed by the Jake tool, making the Lever tool the safest method to remove a manhole cover. The exoskeleton improved the Lever tool’s results even further by reducing the lower back muscle activity and improving the REBA scores while performing the task. However, if a worker prefers a Jake tool over a Lever tool, wearing the back-support exoskeleton can help reduce the ergonomic risk and load on the lower back muscles during the task.

This study demonstrated that passive exoskeletons could reduce lower back muscle strain and improve posture in manhole cover removal tasks, with the most significant effects observed when using the Jake tool. These findings suggest that passive exoskeletons could serve as a viable ergonomic intervention for reducing musculoskeletal strain in physically demanding jobs, such as utility maintenance and construction.

Future research should investigate the long-term impact of exoskeleton use, worker adaptation over time, and how training programs can improve user experience. Additionally, studies should compare passive and active exoskeletons across various industrial tasks to assess their respective advantages and limitations. Expanding experiments to diverse worker populations and real-world environmental conditions will be essential for understanding the broader applicability of exoskeleton technology in industry.

## Figures and Tables

**Table 1:** Summary of all the trial conditions during the study.

Trial	Tool	Exoskeleton Mode	Cover Weight
1	Manual	Without exoskeleton	18.1 kg (40 lbs.)
2		Instant mode	18.1 kg (40 lbs.)
3		Standard mode	18.1 kg (40 lbs.)
4	Jake	Without exoskeleton	56.7 kg (125 lbs.)
5		Instant mode	56.7 kg (125 lbs.)
6		Standard mode	56.7 kg (125 lbs.)
7	Lever	Without exoskeleton	56.7 kg (125 lbs.)
8		Instant mode	56.7 kg (125 lbs.)
9		Standard mode	56.7 kg (125 lbs.)

## Data Availability

The generated datasets are available upon request to the corresponding author.

## Supplementary Materials

The following supporting information can be downloaded at https://www.mdpi.com/article/10.3390/s25072027/s1. Samples of raw EMG data collected at the job site.
