# Supplementary material for: Assessing Effectiveness of Passive Exoskeletons and Tool Selection on Ergonomic Safety in Manhole Cover Removal
Source: Sensors (Basel). 2025 Mar 24;25(7):2027. doi: 10.3390/s25072027 (PMC11991122; doi:10.3390/s25072027)
Supplement: Supplementary file 1 [file sensors-25-02027-s001.zip › sensors-3439712-supplementary.pdf]

sensors

MDPI

Supplementary Materials: Samples of raw EMG data collected in job site

Figures S1-S3 present the raw EMG signals of three subjects under different task conditions. Each figure corresponds to a specific task: Manual manhole cover removal without wearing the exoskeleton, manhole cover removal using the Jake tool while wearing the exoskeleton, and manhole cover removal using the Lever tool without wearing the exoskeleton. The plots display the unprocessed EMG signals from 16 muscle groups, including upper limb, trunk, and lower limb muscles.

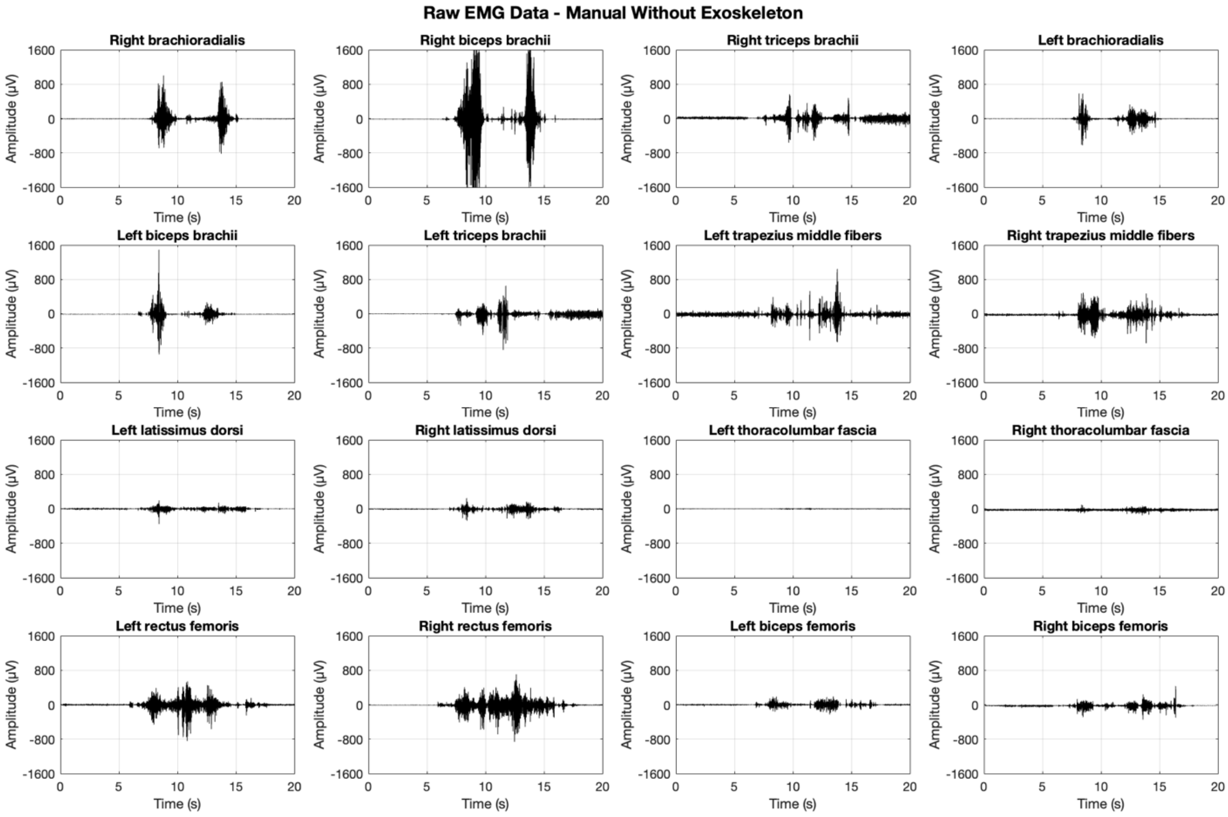

Figure S1. Raw EMG signals of a participant performing manual manhole cover removal without wearing the exoskeleton. The plots display unprocessed EMG signals from 16 muscle groups, including upper limb, trunk, and lower limb muscles.

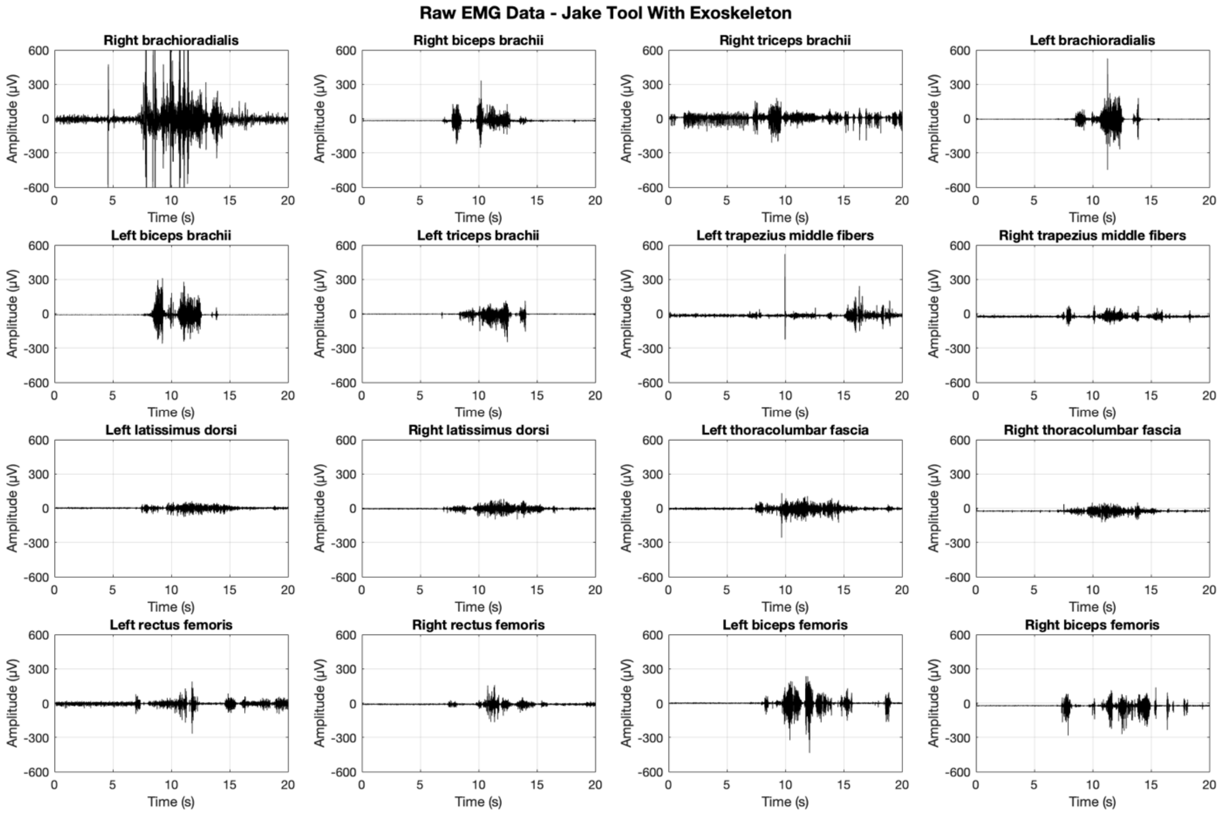

Figure S2. Raw EMG signals of a participant performing manhole cover removal using the Jake tool while wearing the exoskeleton. The plots display unprocessed EMG signals from 16 muscle groups, including upper limb, trunk, and lower limb muscles.

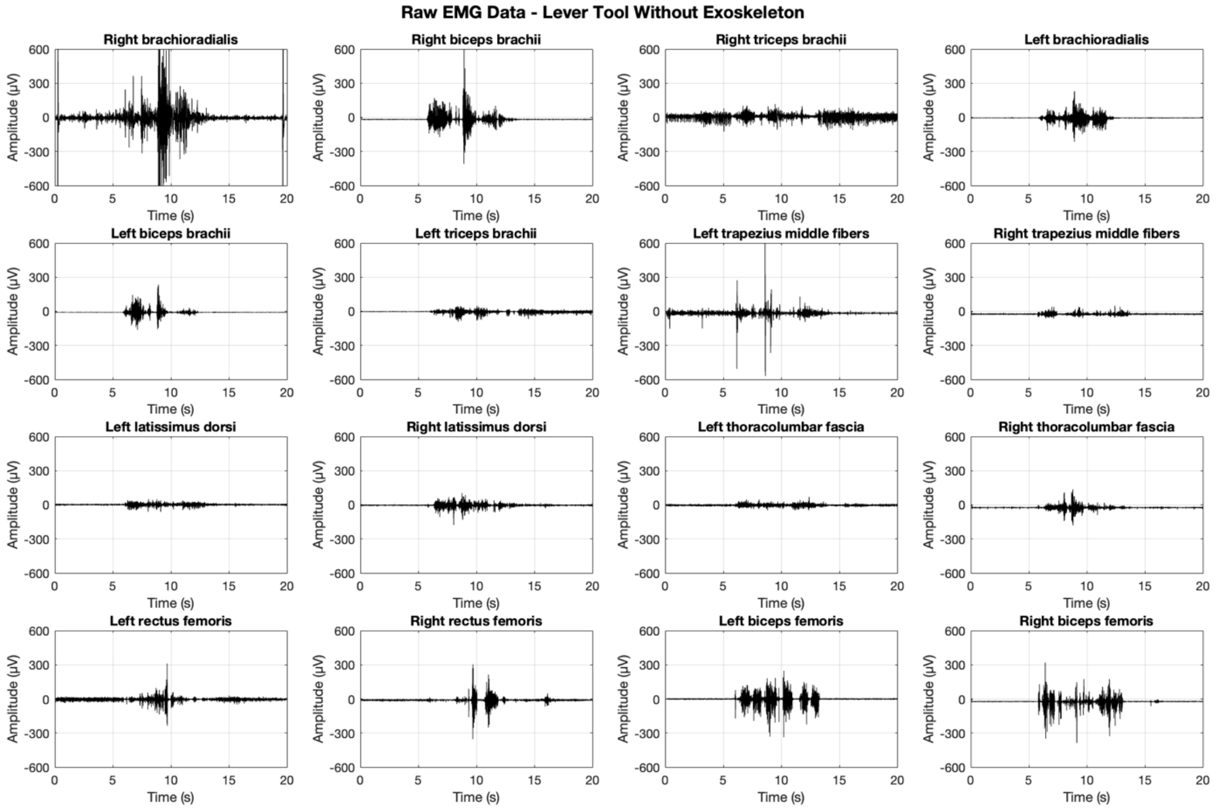

Figure S3. Raw EMG signals of a participant performing manhole cover removal using the Lever tool without wearing the exoskeleton. The plots display unprocessed EMG signals from 16 muscle groups, including upper limb, trunk, and lower limb muscles.
